# Pericardial Tamponade following Asymptomatic SARS-CoV-2 Infection: A Diagnostic Journey

**DOI:** 10.1155/2022/1332844

**Published:** 2022-09-22

**Authors:** Christian Birner, Matthias Gasche, Rainer Voisard, Christian Neumann

**Affiliations:** Joint Medical Service (Germany), Department of Internal Medicine, Military Hospital Ulm, 89081 Oberer Eselsberg 40 Ulm, Germany

## Abstract

*Background*. Pericardial tamponade is a known life-threatening condition rarely reported in COVID-19 but has not been reported following asymptomatic SARS-CoV-2 infection. Its pathomechanism is still elusive. *Case Summary*. We report the case of a 66-year-old man with progressive shortness of breath and leg swelling due to new-onset heart failure and pericardial tamponade following asymptomatic SARS-CoV-2 infection. Ultrasound-guided placement of a pericardial drainage led to significant improvement of symptoms and revealed an exudative effusion. Throughout the diagnostic process, we were confronted with a systemic inflammatory syndrome suspicion of an induced autoimmune condition. After steroid pulse therapy and oral anticoagulation for subclavian vein thrombosis, the patient was discharged and followed in our outpatient clinic. *Discussion*. Patients with asymptomatic SARS-CoV-2 infection are at risk for developing life-threatening complications. Induced autoimmune conditions could be a potential explanation for late-onset pericardial tamponade in this population. A multimodal imaging approach is crucial in the diagnosis and characterization of cardiac inflammation. An interdisciplinary approach is essential. Awareness of uncommon cardiac complications following a SARS-CoV-2 infection is crucial for the initial assessment and the appropriate treatment of these patients.

## 1. Introduction

Pericardial tamponade is a known life-threatening condition rarely reported in COVID-19 but has not been reported following asymptomatic SARS-CoV-2 infection. We report the case of a 66-year-old man with myopericarditis, pericardial tamponade, and polyserositis within five weeks after asymptomatic infection with SARS-CoV-2. Throughout the diagnostic process, we were confronted with a systemic inflammatory syndrome suspicious of an induced autoimmune condition. This report highlights a dedicated diagnostic workup by multimodal cross-sectional imaging studies. Induced autoimmune conditions could be a potential explanation for late-onset pericardial tamponade in this population.

## 2. Case Presentation

**Figure 1:**
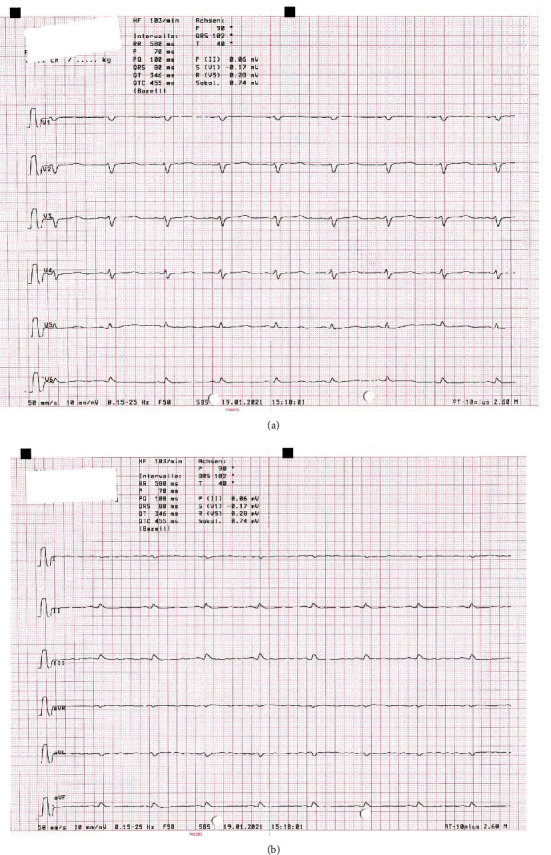
(a, b) Electrocardiogram (50 mm/s) showing low-voltage morphology, tachycardic sinus rhythm (103 bpm), and repolarization abnormalities.

A 66-year-old man without any known comorbidities or allergies presented to our emergency department with ten-day progressive shortness of breath without chest pain, abdominal distension, decreased effort tolerance, and 12 kg weight gain within the last ten days. The first assessment showed an afebrile patient (36.5°C) with sinus tachycardia (108 bpm), hypertension (153/110 mmHg), respiratory rate 15/min, SpO2 87% on room air, signs of low voltage in ECG (Figures 1(a) and 1(b)), anasarca, ascites, pleura effusion, and a massive pericardial effusion on ultrasound evaluation.

39 days prior, the unvaccinated patient was tested positive for SARS-CoV-2 (by RT-PCR) as part of a health care workers' screening. Prior to the infection, the patient was healthy and fit, doing cycling regularly. During the infection, the patient remained asymptomatic. On admission, the patient was tested negative for influenza, respiratory syncytial virus, and SARS-CoV-2 by RT-PCR. SARS-CoV-2-IgG was positive. Laboratory assessment (Table 1) showed leucocytosis with absolute lymphopenia, elevated C-reactive protein, and IL-6 indicating an inflammatory process. Furthermore, biomarkers of myocardial damage were elevated while NT-pro-BNP was within normal limits. We suspected concomitant shock physiology as serum lactate was elevated (3.3 mmol/l).

**Figure 2:**
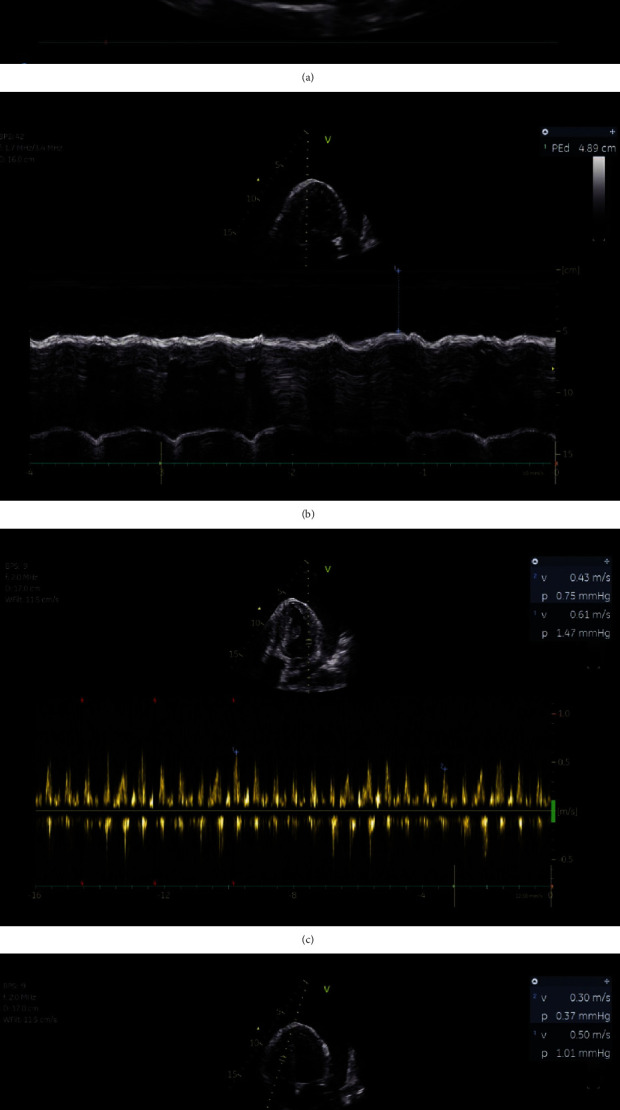
Initial transthoracic echocardiography indicating pericardial tamponade. (a) Apical 4-chamber view with large circumferential pericardial effusion. (b) Apical 4-chamber view (M-mode) with 4.89 mm pericardial effusion end diastolic. (c) Mitral inflow respiratory variation > 25% and (d) tricuspid inflow respiratory variation > 40% by PW-Doppler indicating tamponade morphology; scale and velocity settings are not optimal due to intensive care unit bedside echocardiography.

A CT-thorax scan showed bilateral lower lobe collapse consolidations, large bilateral pleural effusion, pericardial effusion, and ground-glass opacification in the left upper lobe. Dedicated echocardiography (supplemental files, video 1) revealed reduced left and right ventricular function, collapse of the left and right atrium, and a massive circumferential pericardial effusion (48.9 mm apical) suggesting echocardiographic tamponade (Figure 2). We suspected new-onset heart failure and echocardiographic pericardial tamponade. The patient was admitted to our medical intensive care unit for noninvasive ventilation and high-flow nasal cannula oxygen therapy for one day.

**Figure 3:**
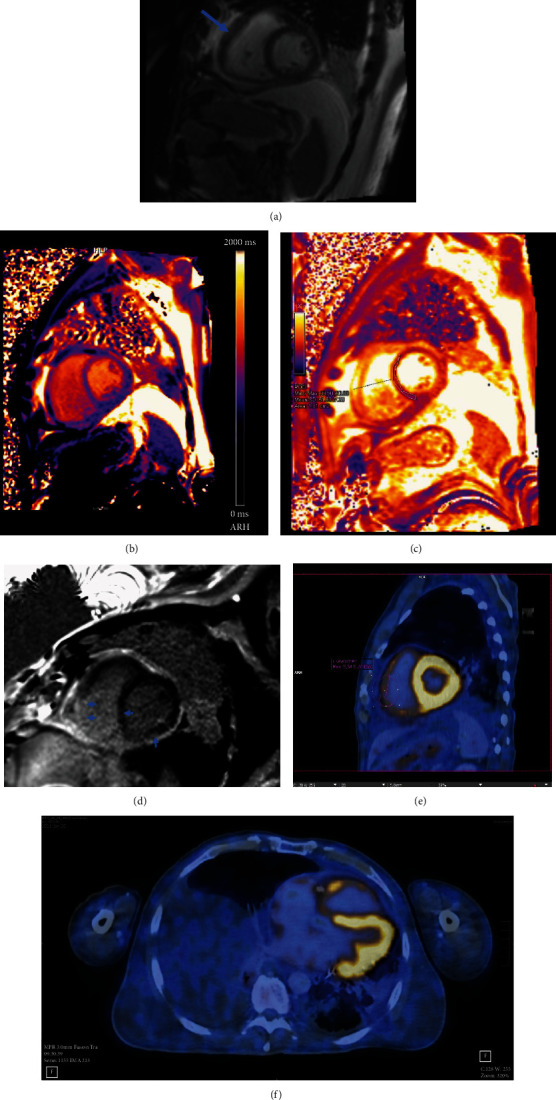
Cross-sectional studies confirmed myocardial inflammation. (a–d) Cardiac magnetic resonance imaging showing right ventricular hypertrophy (a, SSFP cine, ∗), increased T1 relaxation time (septal: 1062 ms, LV posterior wall: 1118 ms) and ECV of 33% (b, T1 map), increased T2 relaxation time to 52 ms septal and 60 ms in LV posterior wall (c, T2 map) and right ventricular, diffuse septal and left ventricular posterior wall late gadolinium enhancement (c, PSIR LGE, arrows) indicating myocarditis and diffuse biventricular fibrosis. (e, f) Positron emission tomography with computed tomography showing FDG uptake in the right ventricular myocardium (SUVmax 5.3), left ventricular uptake was within normal limits.

Due to pericardial tamponade, a step-by-step approach was initiated with pericardiocentesis first for symptomatic therapy and assessing the most common etiologies of pericardial effusion. An ultrasound-guided pericardial drainage was placed, and 850 ml of serosanguinous fluid was removed. Fluid analysis tested negative by RT-PCR for SARS-CoV-2, revealing high-level LDH and protein indicating an exudative process; microbiology (including mycobacterial and fungal culture) remained negative as well as serology for cardiotropic pathogens. Interferon-y (QuantiFERON) testing was positive. After pericardiocentesis, symptoms improved. Cytologic evaluation of the pericardial fluid was suspicious for high malignant lymphoma. Further flow cytometry and immunophenotyping excluded a monoclonal cell population as well as PET-CT showed no pathologic extracardiac FDG uptake, which could have indicated a highly proliferative tumor mass or active tuberculosis. Pleurocentesis was performed for diagnostic purpose and symptomatic treatment. The exudative fluid showed no evidence of malignancy or infection but indicating polyserositis. After removal of at least 1850 ml in total over 9 days, echocardiography showed minimal residual effusion and the drainage was removed. Left ventricular function recovered, but the right ventricular global function was still impaired. Echocardiography revealed hypertrophy of the free right ventricular wall. Cross-sectional studies (Figure 3) confirmed myocardial inflammation indicating myocarditis with diffuse biventricular fibrosis. Cardiac catheterization excluded occlusive coronary artery disease.

A duplex and compression sonography of the upper extremities was performed due to new-onset pain in both arms. It confirmed thrombosis of the left subclavian vein and symmetrical thrombophlebitis of the cephalic vein. An extensive vasculitis and collagenosis screening revealed elevated ANA titer (1 : 100) with high positivity for Ro52, borderline positivity for nRNP/Sm, negative anti-ds-DNA, anti-Sm-antibodies, and normal complement C3/C4. Direct Coombs test (anti-Fy(b)) was positive without evidence of hemolysis. An antiphospholipid syndrome evaluation revealed a slight elevated anti-cardiolipin-IgM titer. Serology for hepatitis A, B, C, and E and HIV was negative. Urine analysis and sediment showed no evidence of concomitant nephropathy. Past medical history was negative for joint pain, skin rash or mucosal lesions, alopecia, and neuropsychiatric abnormalities. Although ACR/EULAR criteria for SLE were negative, 100 mg oral prednisolone was administered for seven days in the set of acute myopericarditis and suspected autoimmune etiology. Within the next three weeks, the patient recovered slowly with loss of about 15 kg along with diuretics and guideline-directed medical heart failure therapy. Left and right ventricular function improved. The elevated liver enzymes had normalized along the improvement of cardiac function suggesting congestive hepatopathy. The patient was discharged after 23 days and followed in our outpatient clinic.

Two weeks later, the patient reported an improvement of his physical performance without any complaints. The laboratory assessment revealed a rise in the ANA titer up to 1 : 320 with persisting high positivity for Ro52 and a positive RT-PCR for SARS-CoV-2 in a nasopharyngeal swab with a cycle threshold of 35.5 indicating low viral shedding. Echocardiography showed a persisting moderate reduced right ventricular function and a pericardial effusion of 8 mm around the right ventricle. A rheumatologic examination was intended but is still pending due to patient's concerns. At a six-month telephone follow-up, the patient was asymptomatic and again at his prior exercise capacity but refused any further medical consultation.

## 3. Discussion

We report the case of a healthy 66-year-old male patient with myopericarditis, pericardial tamponade, and polyserositis within five weeks after asymptomatic infection with SARS-CoV-2.

A negative SARS-CoV-2 PCR and a positive IgG titer at admission indicated a postinfection condition on the background of a positive RT-PCR 39 days prior. At follow-up two weeks after discharge, SARS-CoV-2 PCR was positive with a cycle threshold of 35.5. Repositive tests by RT-PCR are very common in recovered COVID-19 patients. The reason is still unclear, but false RT-PCR results, intermittent virus shedding, viral reactivation, or reinfection with another SARS-CoV-2 strain are currently being discussed [1]. In our institution, sequencing was not routine at this point of time; furthermore, genetic sequencing of the first positive swab was not available and the high cycle threshold on the follow-up PCR indicated no infectivity.

Hypertension is not a characteristic feature of tamponade but is not uncommon in subacute pericardial tamponade [2, 3]. Symptoms, diagnostic findings, and point-of-care echocardiography indicated cardiac tamponade in our patient.

We used a step-by-step approach for excluding the most common etiologies for pericardial effusion, which are cancer (10-25%), pericarditis and infectious causes (15-30%), and connective tissue diseases (5-15%), while tuberculosis is the dominant etiology in developing countries (>60%). Nevertheless, up to 50% still remain idiopathic [4].

Initial cytology findings of the pericardial fluid were suspicious for aggressive lymphoma. Atrial and right heart involvement in our cross-sectional studies may be suggestive for cardiac lymphoma as malignant pericardial effusions can result in tamponade, but primary lymphoma of the heart is rare. Common imaging features in these cases are ill-defined infiltrative masses, tumor extension along the epicardial surfaces, and pericardial thickening [5]. Saab et al. examined 419 pericardial effusion specimens obtained from 364 patients. Only 15% of the specimens were positive for malignancy. The most common primary malignancies were breast and lung cancers, and only 3 of 51 patients had a hematologic malignancy. The sensitivity of cytology for diagnosing malignancy was 92.1%, and false-negative rate was 14.7% [6]. In our case, PET-CT showed no evidence for malignancy and cardiac MRI findings were conclusive for diffuse myocarditis. Immunophenotypisation of the pericardial fluid and an unremarkable follow-up at six months finally excluded this rare diagnosis.

An active extrapulmonary tuberculosis was discussed, but neither microscopy nor culture or PET-CT could confirm this hypothesis. On the other hand, the professional background (volunteer in a nursing home) and the positive QuantiFERON test are still suggestive and should prompt further diagnostic workup if disease progress is suspected, but the uneventful time course argued against an active disease process.

There was no evidence for any common infectious myocarditis, but SARS-CoV-2 infection remained a potential cause.

Cardiac involvement is a salient feature in COVID-19 and is associated with a worse outcome. Pericardial tamponade is a known but rarely reported complication of myopericarditis in COVID-19. Its underlying pathomechanism is still elusive [7]. Farina et al. reported a case of cardiac tamponade 23 days after a positive SARS-CoV-2 nasopharyngeal swab in early 2020. The serohemorrhagic pericardial fluid was tested positive for SARS-CoV-2 by RT-PCR while the second nasopharyngeal swab was negative. They suspected pericardial persistence and a possible direct virus involvement [8]. Furthermore, Garcia-Cruz et al. reported another case of cardiac tamponade with hemorrhagic pericardial effusion secondary to COVID-19. For the first time, they documented changes compatible with acute pericarditis in a pericardial tissue sample with the presence of viral particles in a blood vessel and the interstitium by electron microscopy [9].

To our knowledge, there are only two cases in literature reporting asymptomatic SARS-CoV-2 infection with pericardial tamponade [10, 11]. One had no proven echocardiographic tamponade [10]; the other had upper respiratory tract infection symptoms and was thus not asymptomatic [11]. None of them revealed autoimmune laboratory abnormalities; both were drained about 700 ml bloody pericardial fluid.

However, in contrast to both cases, our patient revealed noticeable increased autoimmune laboratory abnormalities. Berger and Volc proposed a model for viral-induced autoimmunity by type I interferon production and reported an increased frequency of lupus anticoagulant, ANA, anti-erythrocyte antibodies, SSA/Ro, and anti-cardiolipin antibodies in patients with COVID-19 [12]. Thus, the question arose if these laboratory findings are unspecific parainfectious findings, directly associated with COVID-19 or initial signs of an autoimmune disease, in particular systemic lupus erythematosus (SLE).

There are two reported cases of COVID-19 associated tamponade with elevated ANA titer; one was diagnosed with SLE with possible antiphospholipid syndrome [13], and the other one had no signs of myocarditis and showed full recovery in a four-week follow-up [14]. Both presented with symptoms of upper respiratory tract infection. Our patient's demographics, symptoms, and diagnostic findings were not meeting the ACR/EULAR criteria for SLE but matched the Systemic Lupus International Collaborating Clinics (SLICC) classification criteria 2012, supporting the diagnosis of SLE with primary lupus myocarditis and possible antiphospholipid syndrome.

A multicentric lupus myocarditis case series reported myocarditis as the first manifestation of SLE in 58.6% of patients and endomyocardial biopsy (EMB) was low yield in the diagnostic process [15]. In our case, we refrained from EMB due to patient's concerns, fast clinical recovery, and diffuse septal LGE and thus low probability for positive histology. On the other hand, subclavian vein thrombosis and cephalic vein thrombophlebitis may reflect a hypercoagulatory state in the context of COVID-19. As Shah et al. suggested, it would be worthwhile to closely follow those patients with positive autoantibodies [16], because reports of COVID-19 mimicking or precipitating rheumatic musculoskeletal diseases points towards a possibility of persisting intermediate to long-term immune dysregulation.

We are aware that despite the extensive workup, the origin of myopericarditis in our case remains unclear particularly due to the lacking histologic evidence. Regardless of this, a direct or indirect association with SARS-CoV-2 still remained a possible candidate in our diagnostic journey as other differential diagnoses were ruled out and disease progression occurred in the context of a recent asymptomatic infection. Further studies are needed to show whether asymptomatic SARS-CoV-2-infected patients are at risk for potential life-threatening sequelae.

## 4. Conclusion

The key feature of this case was the diagnostic process in a subacute to late-onset myopericarditis following an asymptomatic SARS-CoV-2 infection and the differentiation between directly linked infectious organ damage, malignancy, and a triggered autoimmune condition. These patients need close follow-up, and an interdisciplinary approach is essential. Awareness of cardiac complications following a SARS-CoV-2 infection is crucial in the diagnostic and therapeutic approach. Due to millions of patients infected in the COVID-19 pandemic, this diagnostic dilemma will be challenging in the future.

## Figures and Tables

**Table 1:** Laboratory assessment at admission and follow-up.

	Reference range	Admission	Follow-up
Hemoglobin	13.5–18.0 g/dl	13.5	10.1
Leucocytes	4.0–9.0/nl	12.5	5.9
Lymphocytes	1.1–4.0/nl	0.38	0.77
Platelet count	150–450/nl	195	260
Serum-creatinine	0.55–1.10 mg/dl	0.68	0.47
Serum-urea	16.6–48.5 mg/dl	82.6	27.8
CRP	<0.5 mg/dl	9	1
Interleukin-6	<7 pg/ml	115	—
PCT	mg/dl	0.35	—
NT-pro-BNP	<879 pg/ml	159	762
hsTropT	<0.014 ng/ml	0.026	0.037
LDH	135–225 U/l	483	221
D-dimer	<0.70 mg/l FEU	10.54	1.52
ESR (1 h)	<21 mm/h		56
GOT	<50 U/l	280	14
GPT	<50 U/l	391	12
Bilirubin	<1.20 mg/dl	2.63	0.74
ANA (IFT)	<1 : 100	1 : 100	1 : 320
ANCA (IFT)	<1 : 10	Negative	Negative
Ro52		High positivity	High positivity
nRNP/Sm		Borderline positivity	Negative
SARS-CoV-2 RT-PCR		Negative	Positive
SARS-CoV-2 ct		—	35.5
SARS-CoV-2-IgG		Positive	—
Adenovirus (ELISA)		IgG/IgM negative	—
HHV-6 (IFT)		IgG positive, IgM negative	—
CMV (CMIA)		IgG/IgM negative	—
Parvovirus B19 (CLIA)		IgG/IgM negative	—

## Additional Points

*Learning Points*. (1) Awareness of cardiac complications following a SARS-CoV-2 infection is crucial in the diagnostic and therapeutic approach. (2) Pericardial tamponade is a rare initial presentation in patients with COVID-19 but should be considered as a potential cause of shock. (3) A multimodal imaging approach is crucial in the diagnosis and characterization of cardiac inflammation. (4) Post viral-induced autoimmune phenomena may play a role in late onset pericardial effusions. (5) Echocardiography is a mainstay in rapid bedside diagnosis of pericardial tamponade.

## Supplementary Materials

Supplementary Materials Video 1: Echocardiography on admission indicating pericardial tamponade.
